# Cardiac Angiosarcoma: A Diagnostic and Therapeutic Challenge

**DOI:** 10.1002/kjm2.70044

**Published:** 2025-05-08

**Authors:** Kang Ying Tan, Yi‐Chang Liu

**Affiliations:** ^1^ Division of Hematology and Oncology, Department of Internal Medicine Kaohsiung Medical University Hospital, Kaohsiung Medical University Kaohsiung Taiwan; ^2^ Faculty of Medicine, College of Medicine, Kaohsiung Medical University Kaohsiung Taiwan; ^3^ Cellular Therapy and Research Center Kaohsiung Medical University Hospital Kaohsiung Taiwan

Dear Editor,

1

Primary cardiac angiosarcoma is a rare and highly aggressive neoplasm, accounting for less than 0.1% of all cardiac tumors. Due to the difficulty of approaching it, timely diagnosis and management are challenging. Here we report a case of primary cardiac angiosarcoma; the diagnosis was impressed after a series of investigations.

A 42‐year‐old woman presented with progressive dyspnea for 4 months. Initial investigations revealed pericardial and pleural effusions. Laboratory testing showed elevated anti‐Ro antibodies and positive anti‐connective tissue disease (CTD) serologies, raising suspicion for autoimmune disease‐related effusions. Empirical treatment with corticosteroids and leflunomide was initiated; however, no clinical improvement was observed. Repeat thoracentesis revealed atypical cells, and subsequent computed tomography (CT) imaging identified a mass involving the right atrium and pericardium (Figure 1A). Positron emission tomography‐computed tomography (PET‐CT) demonstrated fluorodeoxyglucose (FDG) uptake in the cardiac sinus and mediastinal lymph nodes (Figure 1B,C). Due to the patient's poor performance status and signs of advanced heart failure, cardiac biopsy was not feasible. Further cytologic analysis of pleural fluid supported the diagnosis of angiosarcoma, with immunohistochemistry positive for cytokeratin (CK), CD31, and ERG (Figure 1D–F).

**FIGURE 1:**
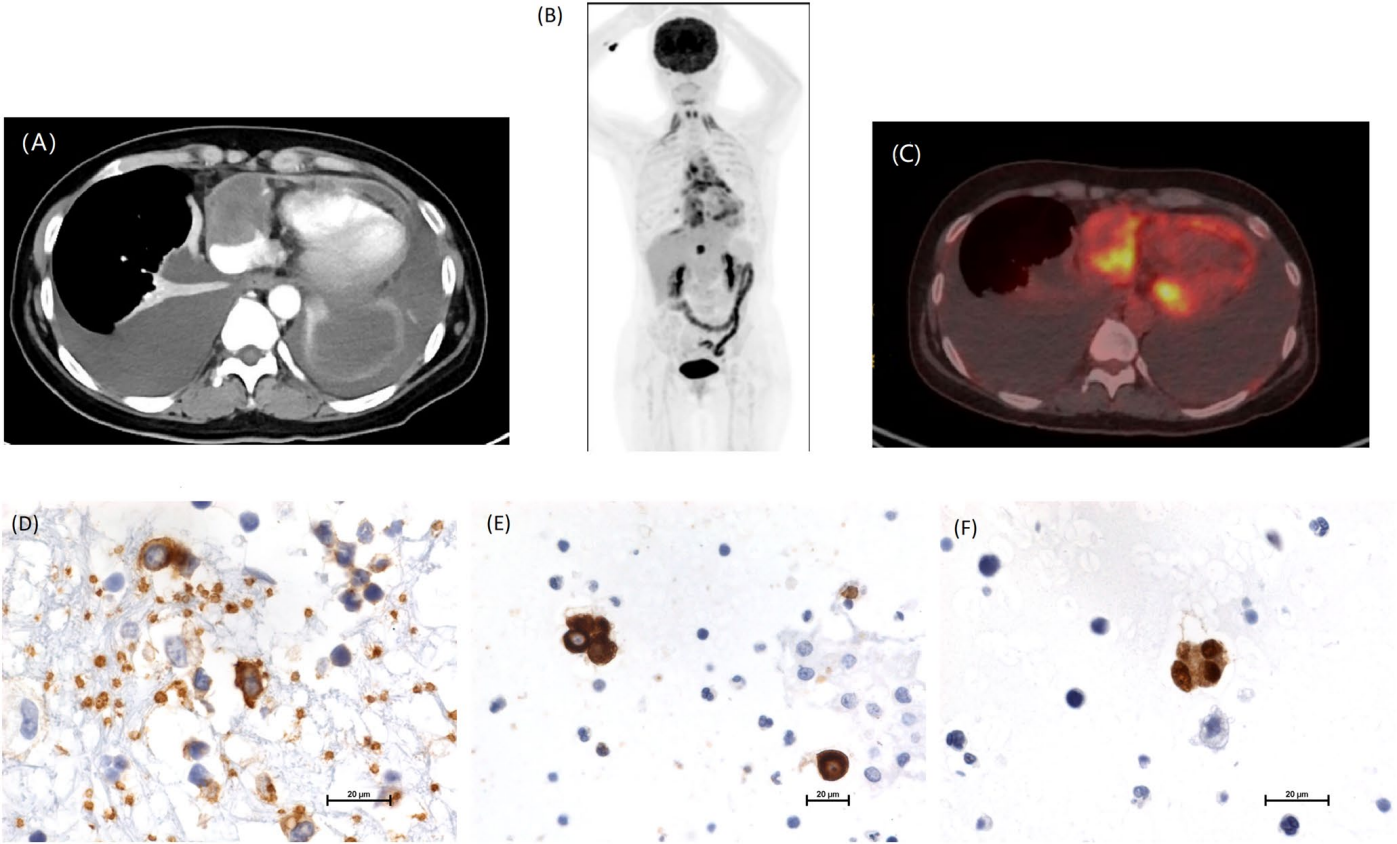
Computed tomography (CT) showed a tumor involving the right pericardium and right atrium (A). Positron emission tomography (PET) scan showed cardiac sinus infiltration and fluorodeoxyglucose (FDG) uptake in mediastinal lymph nodes (B and C). Pleural Fluid cytology immuohistochemical stains showed CK(+) (D), CD31(+) (E) and ERG(+) (F).

The patient received chemotherapy with doxorubicin and ifosfamide, but severe pancytopenia complicated by pneumonia and septic shock developed later. The treatment was then switched to cabozantinib and nivolumab; however, no tumor shrinkage was observed, with worsening effusions. A final attempt with gemcitabine and docetaxel was interrupted by respiratory failure, and the patient passed away 8 months after the initial symptom onset.

This case underscores the diagnostic challenge posed by cardiac angiosarcomas, especially in the context of overlapping autoimmune features. While imaging and cytology aid in diagnosis, tissue confirmation remains critical. Currently, no established link between autoimmune disease and cardiac angiosarcoma exists; only sparse pieces of literature focusing on autoimmune phenomena associated with sarcoma are reported [1, 2, 3].

Therapeutic options for unresectable cardiac angiosarcoma are quite limited. Although immunotherapy like cabozantinib and nivolumab combination has shown promise in other angiosarcoma subtypes [4], our patient did not benefit. Tumor location and subtype may influence response, with cardiac sarcomas possibly demonstrating lower immunogenicity [5].

In conclusion, this case highlights the need for early diagnostic interventions in unexplained recurrent pericardial effusion and emphasizes the urgency for developing effective targeted and immunotherapeutic treatments for cardiac angiosarcoma.

## Conflicts of Interest

The authors declare no conflicts of interest.

## Data Availability

The data that support the findings of this study are available from the corresponding author upon reasonable request.
